# Supplementary material for: Keel Bone Damage in Laying Hens—Its Relation to Bone Mineral Density, Body Growth Rate and Laying Performance
Source: Animals (Basel). 2021 May 25;11(6):1546. doi: 10.3390/ani11061546 (PMC8228274; doi:10.3390/ani11061546)
Supplement: Supplementary file 1 [file animals-11-01546-s001.zip › Suppl_Table_S1.pdf]

Table S1. Percentage of dissected keel bones with/without several damages in hens kept in the small and large compartments of the floor housing system of the first generation.

	BLA		L68		WLA		R11	
Compartment size	small n = 31	large n = 40	small n = 37	large n = 42	small n = 36	large n = 37	small n = 47	large n = 48
Percentage of keel bones with/without deformities[1]								
Score 4	77.42	65.00	89.19	76.19	44.44	37.84	53.19	41.67
Score 3	22.58	25.00	10.81	19.05	41.67	35.14	42.55	52.08
Score 2	0	10.00	0	4.76	13.89	27.03	4.26	6.25
Significance	A	A	A	A	A	A	A	A
Direction of keel bone deformity[2]								
Score 0	0	0	0	0	0	0	4.35	0
Score 1	100	78.57	100	80.00	75.00	56.52	86.96	78.57
Score 2	0	21.43	0	20.00	25.00	43.48	8.70	21.43
Significance	A	A	A	A	A	A	A	A
Percentage of keel bones with/without fractures[3]								
Score 0	51.61	20.00	91.89	61.90	38.89	16.22	80.43	62.50
Score 1	48.39	80.00	8.11	38.10	61.11	83.78	19.57	37.50
Significance	A	B	A	B	A	B	A	B
Percentage of numbers of fractures in the caudal third of fractured keel bones								
1	42.86	35.48	66.67	50.00	52.17	37.50	66.67	47.06
2	50.00	22.58	0	14.29	26.09	25.00	33.33	35.29
3	7.14	22.58	33.33	21.43	13.04	12.50	0	17.65
≥4	0	19.35	0	14.29	8.70	25.00	0	0
Significance	A	B	A	A	A	A	A	A

[1] Scoring system: 4 = no deformity, 3 = slight deformity, 2 = moderate to severe deformity.

[2] Scoring system: 0 = sagittal, 1 = transverse, 2 = sagittal and transverse.

[3] Scoring system: 0 = fracture absent, 1 = fracture present.

A,B: Overall frequencies within a row and layer line with no common letter differ significantly at $p < 0.05$.
