# Supplementary material for: Keel Bone Damage in Laying Hens—Its Relation to Bone Mineral Density, Body Growth Rate and Laying Performance
Source: Animals (Basel). 2021 May 25;11(6):1546. doi: 10.3390/ani11061546 (PMC8228274; doi:10.3390/ani11061546)
Supplement: Supplementary file 1 [file animals-11-01546-s001.zip › Suppl_Table_S2.pdf]

Table S2. Percentage of dissected keel bones with/without several damages in hens kept in the small and large compartments of the floor housing system of the second generation.

	BLA		L68		WLA		R11	
Compartment size	small n = 47	large n = 45	small n = 48	large n = 42	small n = 32	large n = 42	small n = 48	large n = 48
Percentage of keel bones with/without deformities[1]								
Score 4	82.98	53.33	93.75	88.10	75.00	38.10	81.25	60.42
Score 3	17.02	28.89	6.25	11.90	18.75	35.71	14.58	31.25
Score 2	0	17.78	0	0	6.25	26.19	4.17	8.33
Significance	A	B	A	A	A	B	A	A
Direction of keel bone deformity[2]								
Score 0	0	0	0	0	0	0	0	0
Score 1	55.56	33.33	100	80.00	75.00	46.15	44.44	68.42
Score 2	44.44	66.67	0	20.00	25.00	53.85	55.56	31.58
Significance	A	A	A	A	A	A	A	A
Percentage of keel bones with/without fractures[3]								
Score 0	55.32	26.67	72.92	61.90	46.88	9.52	68.75	31.25
Score 1	44.68	73.33	27.08	38.10	53.13	90.48	31.25	68.75
Significance	A	B	A	A	A	B	A	B
Percentage of numbers of fractures in the caudal third of fractured keel bones								
1	66.67	42.42	76.92	66.67	52.94	26.32	73.33	69.70
2	28.57	30.30	23.08	26.67	41.18	23.68	20.00	24.24
3	0	12.12	0	0	5.88	18.42	6.67	6.06
≥4	4.76	15.15	0	6.67	0	31.58	0	0
Significance	A	A	A	A	A	B	A	A

[1] Scoring system: 4 = no deformity, 3 = slight deformity, 2 = moderate to severe deformity.

[2] Scoring system: 0 = sagittal, 1 = transverse, 2 = sagittal and transverse.

[3] Scoring system: 0 = fracture absent, 1 = fracture present.

A,B: Overall frequencies within a row and layer line with no common letter differ significantly at $p < 0.05$.
