# Supplementary material for: Keel Bone Damage in Laying Hens—Its Relation to Bone Mineral Density, Body Growth Rate and Laying Performance
Source: Animals (Basel). 2021 May 25;11(6):1546. doi: 10.3390/ani11061546 (PMC8228274; doi:10.3390/ani11061546)
Supplement: Supplementary file 1 [file animals-11-01546-s001.zip › Suppl_Table_S3.pdf]

Table S3. The effects of generation, layer line, housing system and their interactions on the results of time-dependent individual growth data fitted to the Gompertz function.

**Effect**	a		b		c	
	F Value	*p*- Value	*F* Value	*p*- Value	*F* Value	*p*- Value
Generation (G)	17.13	<0.001	15.99	<0.001	14.54	<0.001
Layer line (LL)	154.3	<0.001	106.7	<0.001	258.8	<0.001
Housing system (HS)	148.55	<0.001	46.38	<0.001	118.8	<0.001
G × LL	1.10	0.351	1.33	0.269	4.48	0.005
G × HS	3.64	0.057	12.74	<0.001	3.34	0.068
LL × HS	20.33	<0.001	17.29	<0.001	18.09	<0.001

a = adult body weight (g) of the hen (asymptotic limit) at 69 weeks of age; b = slope of the growth curve; c = point of inflection (weeks)
