# Supplementary material for: Keel Bone Damage in Laying Hens—Its Relation to Bone Mineral Density, Body Growth Rate and Laying Performance
Source: Animals (Basel). 2021 May 25;11(6):1546. doi: 10.3390/ani11061546 (PMC8228274; doi:10.3390/ani11061546)
Supplement: Supplementary file 1 [file animals-11-01546-s001.zip › Suppl_Table_S4.pdf]

Table S4. Least Square Means (LSM), standard errors (SE) and significant differences between the four layer lines within the two housing systems for the results of time-dependent individual growth data fitted to the Gompertz function of the first and second generation, respectively.

Effect		a	b	c
		LSM ± SE	LSM ± SE	LSM ± SE
**Generation**				
1st generation		1667^{B} ± 12.96	0.146^{A} ± 0.001	9.67^{A} ± 0.053
2nd generation		1742^{A} ± 12.87	0.140^{B} ± 0.001	9.38^{B} ± 0.052
**Layer line**				
BLA		1840^{B} ± 18.33	0.148^{B} ± 0.001	8.93^{C} ± 0.075
L68		1941^{A} ± 18.27	0.137^{C} ± 0.001	9.35^{B} ± 0.075
R11		1449^{D} ± 18.03	0.128^{D} ± 0.001	11.25^{A} ± 0.073
WLA		1588^{C} ± 18.41	0.159^{A} ± 0.001	8.58^{D} ± 0.076
**Housing system**				
Floor housing		1754^{A} ± 9.91	0.140^{B} ± 0.001	9.80^{A} ± 0.044
Cages		1656^{B} ± 10.05	0.146^{A} ± 0.001	9.25^{B} ± 0.046
**Generation x Layer line**				
1st generation	BLA	1808^{B} ± 26.19	0.150^{BC} ± 0.002	8.99^{CDE} ± 0.109
	L68	1893^{AB} ± 26.00	0.140^{DE} ± 0.002	9.44^{C} ± 0.107
	R11	1435^{D} ± 25.35	0.128^{F} ± 0.002	11.62^{A} ± 0.103
	WLA	1532^{D} ± 26.11	0.163^{A} ± 0.002	8.63^{E} ± 0.108
2nd generation	BLA	1872^{B} ± 25.66	0.145^{CD} ± 0.002	8.87^{DE} ± 0.104
	L68	1990^{A} ± 25.68	0.133^{EF} ± 0.002	9.26^{CD} ± 0.104
	R11	1463^{D} ± 25.62	0.127^{F} ± 0.002	10.87^{B} ± 0.104
	WLA	1645^{C} ± 25.98	0.155^{AB} ± 0.002	8.53^{E} ± 0.107
**Generation x Housing system**				
1st generation	Floor housing	1724^{B} ± 14.13	0.141^{B} ± 0.001	9.99^{A} ± 0.064
	Cages	1610^{C} ± 14.29	0.150^{A} ± 0.001	9.35^{C} ± 0.066
2nd generation	Floor housing	1784^{A} ± 13.90	0.139^{B} ± 0.001	9.61^{B} ± 0.061
	Cages	1701^{B} ± 14.14	0.142^{B} ± 0.001	9.16^{C} ± 0.064
**Layer line x Housing system**				
BLA	Floor housing	1840^{B} ± 19.91	0.150^{BC} ± 0.002	9.02^{D} ± 0.089
	Cages	1840^{B} ± 20.21	0.145^{CD} ± 0.002	8.84^{D} ± 0.093
L68	Floor housing	1992^{A} ± 19.78	0.133^{E} ± 0.002	9.53^{C} ± 0.088
	Cages	1890^{B} ± 20.12	0.141^{D} ± 0.002	9.17^{D} ± 0.092
R11	Floor housing	1535^{D} ± 19.34	0.123^{F} ± 0.002	11.82^{A} ± 0.085
	Cages	1363^{E} ± 19.90	0.132^{E} ± 0.002	10.68^{B} ± 0.091
WLA	Floor housing	1648^{C} ± 20.23	0.153^{B} ± 0.002	8.85^{D} ± 0.092
	Cages	1529^{D} ± 20.16	0.166^{A} ± 0.002	8.31^{E} ± 0.093

a = adult body weight (g) of the hen (asymptotic limit) at 69 weeks of age; b = slope of the growth curve; c = point of inflection (weeks)

$^{A-E}$ Means within a trait and effect with no common superscript differ significantly at $P < 0.05$
